# New Imidazole-Based *N*-Phenylbenzamide Derivatives as Potential Anticancer Agents: Key Computational Insights

**DOI:** 10.3389/fchem.2021.808556

**Published:** 2022-01-19

**Authors:** M. Shaheer Malik, Reem I. Alsantali, Qazi Mohammad Sajid Jamal, Zaki S. Seddigi, Moataz Morad, Meshari A. Alsharif, Essam M. Hussein, Rabab S. Jassas, Munirah M. Al-Rooqi, Zainularifeen Abduljaleel, Ahmed O. Babalgith, Hatem M. Altass, Ziad Moussa, Saleh A. Ahmed

**Affiliations:** ^1^ Department of Chemistry, Faculty of Applied Sciences, Umm Al-Qura University, Makkah, Saudi Arabia; ^2^ Department of Pharmaceutical Chemistry, College of Pharmacy, Taif University, Taif, Saudi Arabia; ^3^ Department of Health Informatics, College of Public Health and Health Informatics, Qassim University, Al Bukayriyah, Saudi Arabia; ^4^ Department of Environmental Health, College of Public Health and Health Informatics, Umm Al-Qura University, Makkah, Saudi Arabia; ^5^ Department of Chemistry, Faculty of Science, Assiut University, Assiut, Egypt; ^6^ Department of Chemistry, Jamoum University College, Umm Al-Qura University, Makkah, Saudi Arabia; ^7^ Science and Technology Unit, Umm Al-Qura University, Makkah, Saudi Arabia; ^8^ Department of Medical Genetics, Faculty of Medicine, Umm Al-Qura University, Makkah, Saudi Arabia; ^9^ Department of Chemistry, College of Science, United Arab Emirates University, Al Ain, United Arab Emirates

**Keywords:** *N*-phenylbenzamide, imidazole, multicomponent reaction, anticancer activity, computational studies, molecular dynamic simulations, ADME and drug-likeness

## Abstract

An efficient atom-economical synthetic protocol to access new imidazole-based *N*-phenylbenzamide derivatives is described. A one-pot three-component reaction was utilized to provide a series of *N-phenylbenzamide* derivatives in a short reaction time (2–4 h) with an 80–85% yield. The cytotoxic evaluation revealed that derivatives 4e and 4f exhibited good activity, with IC_50_ values between 7.5 and 11.1 μM against the tested cancer cell lines. Computational studies revealed interesting insights: the docking of the active derivatives (4e and 4f) showed a higher affinity toward the target receptor protein than the control. Molecular dynamic simulations revealed that the active derivatives form stable complexes with the ABL1 kinase protein. Moreover, the ADME and drug-likeness of the derivatives reinforced the potential of the derivatives to be taken up for further development as anticancer agents.

## Introduction

**FIGURE 1:**
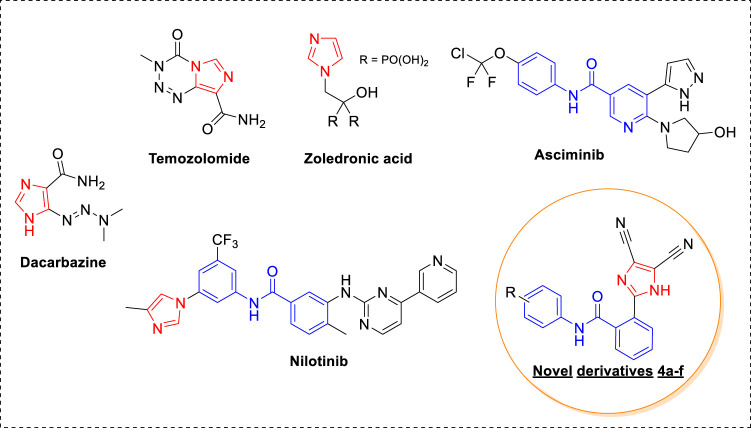
Anticancer agents with imidazole and *N*-phenylbenzamide derivatives.

In recent years, momentous advances in the detection and treatment of cancer have taken place; however, cancer still poses a stiff challenge (Mullard, 2020). This is because of the high cancer incidence rate, resulting from a sedentary and unhealthy lifestyle, and the increase in resistance toward anticancer drugs used in the clinic. Therefore, there is a constant need to develop new anticancer agents. Heterocyclic-based compounds are the backbone of anticancer drug design and discovery (Ali et al., 2015). An imidazole ring with an electron-rich character assists in binding diverse biological receptors at the molecular level, resulting in a plethora of pharmaceutical applications (Zhang et al., 2014). It is a key structural feature in various clinically used anticancer drugs and the development of new anticancer agents (Ali et al., 2017). Dacarbazine, a carbonylimidazole, is used for treating malignant melanoma, and temozolomide, a fused imidazole, is effective against malignant brain tumors (Figure 1) (Mrugala et al., 2010; Narahira et al., 2020). Zoledronic acid, an imidazole with biphosphate functionality, is used in the treatment of multiple myeloma and bone metastasis (Ibrahim et al., 2003). In addition to this, different kinase inhibitors that are used as anticancer drugs contain imidazole rings such as nilotinib and asciminib. Nilotinib is a tyrosine kinase inhibitor effective against chronic myelogenous leukemia (CML) resistant to imatinib (Gleevec), a first-line drug (Sacha and Saglio, 2019). Notably, the U.S. FDA accepted a priority review of asciminib for treating chromic CML this year (Garcia-Gutiérrez et al., 2021). An interesting feature of kinase inhibitors such as imatinib, nilotinib, and asciminib is the presence of *N*-phenyl benzamide derivatives with different heterocycles. Moreover, the substitutions on the benzene ring are in either 1, 3 or 1,4 positions. Considering the importance of imidazole and *N*-phenyl benzamide functionality, we designed new imidazole-based *N*-phenylbenzamide derivatives as potential anticancer agents. The structural design was undertaken on two fronts: the first was to incorporate 1, 2 substitution on the benzene ring, and the second was to conserve the imidazole ring only by excluding pyrimidine and pyridine rings. Additionally, dicyano functionality was introduced into the imidazole ring. It is noteworthy that the nitrile group plays an efficacious role in different nitrile-containing drugs used in the clinic (Fleming et al., 2010). From our previous research endeavors, we synthesized a series of desired new imidazole-based *N*-phenylbenzamide derivatives by using an atom-economical one-pot multicomponent reaction strategy (Shaheer Malik et al., 2019; Hussein et al., 2021; Malik et al., 2021; Malik et al., 2022). The anticancer potential of new *N*-phenylbenzamide derivatives was evaluated against selected human cancer cell lines, and the potent derivatives were subjected to molecular docking to understand binding affinity at the molecular level. Moreover, molecular dynamic simulations were carried out to develop a deeper understanding of the interactions at the receptor level. The molecular descriptors and drug-likeness properties of the derivatives were also studied computationally.

## Materials and Methods

### Chemical Synthesis

The chemicals used in the study were purchased from Sigma Aldrich, and the progress of the reactions was examined by thin-layer chromatography, and UV irradiation was used for visualization. Fourier transform infrared (IR) spectroscopy was done on a VERTEX 70 Bruker instrument by using potassium bromide pellets. ^1^H NMR and ^13^C NMR spectroscopy were carried out on a Bruker DRX-400 spectrometer using DMSO-*d*_6_ and TMS as a solvent and internal standard, respectively. The chemical shifts (d) were reported in ppm, and mass spectra were recorded on the Agilent-LCMS instrument.

#### Procedure for Synthesis of Imidazole Based *N*-Phenylbenzamide Derivatives 4a-j

To a mixture of phthalic anhydride 1 (10 mmol), substituted anilines 2a–j (10 mmol), and 2,3-diaminomaleonitrile 3 (10 mmol) in ethanol (50 ml), 0.5 eq of HCl (0.4 ml) was added. The reaction mixture was refluxed in the round-bottom flask for 2–3 h, and the course of the reaction was monitored by thin layer chromatography. As the reaction completed, 100 ml of water was added, and the resulting mixture was stirred for 20–30 min. Colorless solid separates out, which was collected by suction filtration, washed with water (25 ml), and dried at 60°C under vacuum for overnight. The compound was purified by recrystallization using ethanol to afford the desired *N*-phenylbenzamide derivatives 4a–j with 80–85% yield.

#### 2-(4,5-Dicyano-1H-imidazol-2-yl)-*N*-phenylbenzamide (4a)

Prepared as mentioned in the general procedure of 4a–f from 1 (10 mmol), substituted aniline 2a (10 mmol) and 3 (10 mmol) to provide 4a; melting point: 253–255°C; yield: 85%; IR (KBr) cm^−1^: 3,430 (broad, -NH), 1,651 (C=O); ^1^H-NMR (400 MHz; DMSO-*d*_6_) δ: 6.8–8.0 (m, 9H, Ar-H), 9.8 (s, 1H, -CONH-), 11.0 (s, 1H, -NH-); ^13^C-NMR (100 MHz; DMSO-*d*_6_) δ: 167.1, 153.9, 134.6, 134.5, 131.9, 131.5, 130.3, 130.3, 128.8, 128.0, 127.3, 123.4, 123.3, 119.1, 118.8, 116.5. [M + H^+^]: 314.

#### *N*-(4-Chlorophenyl)-2-(4,5-dicyano-1H-imidazol-2-yl)benzamide (4b)

Prepared as mentioned in the general procedure of 4a–f from 1 (10 mmol), substituted aniline 2b (10 mmol) and 3 (10 mmol) to provide 4b; melting point: 271–273°C; yield: 82%; IR (KBr) cm^−1^: 3,435 (broad, -NH), 1,653 (C=O); ^1^H-NMR (400 MHz; DMSO-*d*_6_) δ: 6.9–8.0 (m, 8H, Ar-H), 9.9 (s, 1H, -CONH-), 11.1 (s, 1H, -NH-); ^13^C-NMR (100 MHz; DMSO-*d*_6_) δ: 166.3, 154.8, 135.7, 134.4, 133.8, 132.8, 130.9, 130.2, 129.8, 128.3, 126.5, 125.5, 124.6, 120.3, 119.8, 117.4; [M^+^]: 347 and [M+2]: 349.

#### 2-(4,5-Dicyano-1H-imidazol-2-yl)-*N*-(4-nitrophenyl)benzamide (4c)

Prepared as mentioned in the general procedure of 4a–f from 1 (10 mmol), substituted aniline 2c (10 mmol) and 3 (10 mmol) to provide 4c; melting point: 292–294°C; yield: 84%; IR (KBr) cm^−1^: 3,445 (broad, -NH), 1,659 (C=O); ^1^H-NMR (400 MHz; DMSO-*d*_6_) δ: 6.8–8.0 (m, 8H, Ar-H), 9.8 (s, 1H, -CONH-), 11.0 (s, 1H, -NH-); ^13^C-NMR (100 MHz; DMSO-*d*_6_) δ: 165.1, 151.9, 134.5, 134.4, 131.6, 131.5, 130.3, 130.3, 128.5, 128.1, 127.3, 123.5, 123.3, 119.1, 118.8, 116.4; [M + H^+^]: 359.

#### 2-(4,5-Dicyano-1H-imidazol-2-yl)-*N*-(p-tolyl)benzamide (4d)

Prepared as mentioned in the general procedure of 4a–f from 1 (10 mmol), substituted aniline 2d (10 mmol) and 3 (10 mmol) to provide 4d; melting point: 251–252°C; yield: 83%; IR (KBr) cm^−1^: 3,433 (broad, -NH), 1,651 (C=O); ^1^H-NMR (400 MHz; DMSO-*d*_6_) δ: 2.8 (s, 3H, -CH_3_-), 6.7–8.0 (m, 8H, Ar-H), 9.7 (s, 1H, -CONH-), 10.9 (s, 1H, -NH-); ^13^C-NMR (100 MHz; DMSO-*d*_6_) δ: 167.1, 155.8, 153.2, 135.6, 135.3, 134.8, 134.6, 134.3, 133.4, 129.4, 128.3, 127.5, 123.6, 122.4, 119.4, 118.5, 116.8, 20.6; [M + H^+^]: 328.

#### 2-(4,5-Dicyano-1H-imidazol-2-yl)-*N*-(4-methoxyphenyl)benzamide (4e)

Prepared as mentioned in the general procedure of 4a–f from 1 (10 mmol), substituted aniline 2e (10 mmol) and 3 (10 mmol) to provide 4e; melting point: 262–265°C; yield: 82%; IR (KBr) cm^−1^: 3,432 (broad, -NH), 1,630 (C=O); ^1^H-NMR (400 MHz; DMSO-*d*_6_) δ: 3.8 (s, 3H, -OCH_3_-), 6.9–8.1 (m, 8H, Ar-H), 9.8 (s, 1H, -CONH-), 10.9 (s, 1H, -NH-); ^13^C-NMR (100 MHz; DMSO-*d*_6_) δ: 167.2, 156.6, 150.4, 134.1, 134.0, 133.8, 133.6, 132.3, 132.1, 130.5, 129.4, 128.6, 125.8, 123.5, 119.6, 118.7, 116.9, 59.3; [M + H^+^]: 344.

#### 2-(4,5-Dicyano-1H-imidazol-2-yl)-*N*-(4-fluorophenyl)benzamide (4f)

Prepared as mentioned in the general procedure of 4a–f from 1 (10 mmol), substituted aniline 2f (10 mmol) and 3 (10 mmol) to provide 4f; melting point: 237–239°C; yield: 84%; IR (KBr) cm^−1^: 3,421 (broad, -NH), 1,632 (C=O); ^1^H-NMR (400 MHz; DMSO-*d*_6_) δ: 7.0–8.0 (m, 8H, Ar-H), 9.9 (s, 1H, -CONH-), 11.2 (s, 1H, -NH-); ^13^C-NMR (100 MHz; DMSO-*d*_6_) δ: 165.3, 157.8, 135.4, 135.0, 134.8, 134.6, 133.3, 133.1, 130.5, 129.5, 128.4, 125.2, 123.1, 119.5, 118.6, 116.0; [M + H^+^]: 332.

#### *N*-(4-Bromophenyl)-2-(4,5-dicyano-1H-Imidazol-2-yl)benzamide (4g)

Prepared as mentioned in the general procedure of 4a–f from 1 (10 mmol), substituted aniline 2g (10 mmol) and 3 (10 mmol) to provide 4g; melting point: 278–280°C; yield: 82%; IR (KBr) cm^−1^: 3,423 (broad, -NH), 1,645 (C=O); ^1^H-NMR (400 MHz; DMSO-*d*_6_) δ: 6.7–8.2 (m, 8H, Ar-H), 9.8 (s, 1H, -CONH-), 11.1 (s, 1H, -NH-); ^13^C-NMR (100 MHz; DMSO-*d*_6_) δ: 166.1, 157.6, 136.3, 135.2, 134.4, 134.1, 133.2, 133.0, 130.4, 129.2, 129.0, 128.3, 127.0, 126.4, 119.0, 118.3; [M^+^]: 391 and [M+2]: 393.

#### 2-(4,5-Dicyano-1H-imidazol-2-yl)-*N*-(2-methoxyphenyl)benzamide (4h)

Prepared as mentioned in the general procedure of 4a–f from 1 (10 mmol), substituted aniline 2h (10 mmol) and 3 (10 mmol) to provide 4h; melting point: 282–284°C; yield: 84%; IR (KBr) cm^−1^: 3,434 (broad, -NH), 1,648 (C=O); ^1^H-NMR, δ (400 MHz; DMSO-*d*_6_; TMS): 3.8 (s, 3H, -OCH_3_-), 6.8–8.2 (m, 8H, Ar-H), 9.9 (s, 1H, -CONH-), 11.0 (s, 1H, -NH-); ^13^C-NMR (100 MHz; DMSO-*d*_6_) δ: 167.6, 151.0, 150.9, 138.7, 137.6, 135.6, 133.2, 130.7, 130.5, 129.0, 128.9, 128.5, 127.3, 123.2, 121.8, 120.2, 115.3, 59.9; [M + H^+^]: 344.

#### 2-(4,5-Dicyano-1H-imidazol-2-yl)-*N*-(o-tolyl)benzamide (4i)

Prepared as mentioned in the general procedure of 4a–f from 1 (10 mmol), substituted aniline 2i (10 mmol) and 3 (10 mmol) to provide 4i; melting point: 279–281°C; yield: 83%; IR (KBr) cm^−1^: 3,426 (broad, -NH), 1,658 (C=O); ^1^H-NMR (400 MHz; DMSO-*d*_6_) δ: 2.8 (s, 3H, -CH_3_-), 6.9–8.1 (m, 8H, Ar-H), 9.8 (s, 1H, -CONH-), 11.0 (s, 1H, -NH-); ^13^C-NMR (100 MHz; DMSO-*d*_6_) δ: 168.6, 151.1, 150.7, 138.9, 137.5, 136.8, 132.3, 130.8, 130.0, 129.5, 129.3, 128.8, 128.5, 127.2, 123.3, 121.9, 120.0, 115.0, 20.9; [M + H^+^]: 328.

#### 2-(4,5-Dicyano-1H-imidazol-2-yl)-*N*-(2-nitrophenyl)benzamide (4j)

Prepared as mentioned in the general procedure of 4a–f from 1 (10 mmol), substituted aniline 2j (10 mmol) and 3 (10 mmol) to provide 4j. Melting point: 283–285°C; Yield: 85%; IR (KBr) cm^−1^: 3,446 (broad, -NH), 1,665 (C=O); ^1^H-NMR (400 MHz; DMSO-*d*_6_) δ: 6.8–8.0 (m, 8H, Ar-H), 9.8 (s, 1H, -CONH-), 11.0 (s, 1H, -NH-); ^13^C-NMR (100 MHz; DMSO-*d*_6_) δ: 165.4, 151.8, 134.4, 134.2, 131.4, 131.1, 130.2, 130.1, 128.4, 128.0, 127.5, 123.0, 122.1, 119.0, 118.5, 116.3; [M + H^+^]: 359.

### Cytotoxicity Assay

In the cytotoxicity assay, three cancer lines, that is, lung cancer (A549), cervical cancer (Hela), and breast cancer (MCF-7) were used. New imidazole-based *N*-phenylbenzamide derivative (4a–j) was investigated for cytotoxic potential by employing tetrazolium dye–based MTT assay (Mosmann, 1983). The individual test derivatives and the positive control, doxorubicin, were solubilized in dimethyl sulfoxide. The cancer cells were cultivated at a density of 2×10^5^ cells in 100 µL of culture medium and grown for a day (24 h) in a 96-well plate. Different concentrations of test compounds were subsequently added to the cells and incubated for 2 days (48 h). After incubation, each well was cleaned with 200 µL of phosphate-buffered saline and incubated with a 10% MTT solution for 2 h at 37°C. A multimode reader at 570 nm (Tecan Infinite 200 PRO, Switzerland) was used to determine the optical density of the solubilized formazan crystals.

### Computational Studies

#### Molecular Modeling

Molecular interactions between ligands 4e, 4f and nilotinib (control) with ABL1 kinase were investigated by the Virtua Drug Docking Server (AutoDockv4) (Morris et al., 2009). The structures of ligands were drawn by the PubChem Sketcher V2.4 with optimized energy, and saved in pdb format (Ihlenfeldt et al., 2009). The three-dimensional crystalline structure of ABL1 kinase was collected from RCSB (PDB IDs: 5MO4) (Wylie et al., 2017). The simulation was carried out in dimensions of grid box points (nx = 20. ny = 20, nz = 20, cx = −44.15, cy = 22.92, cz = −19.18), with 1 Å grid distance. Finally, from the analysis of the output file was analyzed, and the best docking poses were selected.

#### Molecular Dynamics Simulations

The molecular dynamics simulations of ABL1 kinase-4e, ABL1 kinase-4f, and ABL1 kinase–nilotinib complexes were executed for 5 nanoseconds (ns) using the GROMACS tool 2018 version (Van Der Spoel et al., 2005). The required ABL1 kinase (PDB:5MO4) topology file was generated by the *pdb2gmx* module, and CHARMM27 all-atom force field selection was applied. The topology files of ligands 4e and 4f and nilotinib were obtained from the SwissParam server (Zoete et al., 2011). The simulation box was setup by the addition of water and Na^+^ and Cl^−^ ions for stabilization of the system, followed by the minimization of energy. The equilibrium setup of the system (ABL1 kinase-4e, ABL1 kinase-4f, and ABL1 kinase–nilotinib) was done, followed by two-step ensembles of NVT and NPT variables. GORMACS contains several packages; for the ABL1 kinase-4e, ABL1 kinase-4f, and ABL1 kinase–nilotinib MDS analysis, we used gmxrms for root-mean-square deviation (RMSD), gmxrmsf for root-mean-square fluctuation (RMSF), gmx gyrate for the calculation of radius of gyration (Rg), and gmx h-bond for the calculation of the numbers of hydrogen bonds formed during interaction (Kuzmanic and Zagrovic, 2010; Kufareva and Abagyan, 2012). Finally, trajectory files and graphical plots were generated by the XMGRACE program, version 5.1.19, after a successful 5-ns simulation run.

#### ADME and Drug-Likeness Predictions

SwissADME program was used for the computational predictions of ADME and drug-likeness properties of the new derivatives (4a–j). The program is provided by the Swiss Institute of Bioinformatics, Switzerland (Daina et al., 2017).

## Results and Discussions

### Chemistry

In the present investigation, the desired novel imidazole-based *N*-phenylbenzamide derivatives 4a–j were synthesized after optimizing a new one-pot multicomponent synthetic methodology. To realize this, phthalic anhydride 1, aniline 2a, and 2,3-diaminomaleonitrile 3 were selected as substrates for a model multicomponent reaction to obtain the compound 2-(4,5-dicyano-1H-imidazol-2-yl)-*N*-phenylbenzamide 4a. Initially, we scrutinized different solvents like ethanol, methanol, dimethylformamide, and dimethyl sulfoxide at room temperature in the presence of an acidic medium provided by simple hydrochloric acid (Table 1). It was observed that the protic solvents, methanol, and ethanol provided higher yield (around 80% yield) compared to DMF and DMSO (70% yield). However, the reaction time was longer in all the tested solvents, that is, 8 and 12 h (entries 1–4). Therefore, we studied the reactions at elevated temperatures, which caused a dramatic decrease in the reaction time. Under the reflux condition, the solvent ethanol gave the best results, with an 85% yield in 2 h of reaction time (entry 6). In addition to hydrochloric acid, other acids such as phosphoric acid and acetic acid were also investigated (entries 9 and 10). However, hydrochloric acid provided best results under the studied conditions. Moreover, varying the amount of hydrochloric acid resulted in a decrease in yield or an increase in reaction time (entries 11 and 12). The results showed that the three-component reaction (1, 2a, and 3) in the presence of hydrochloric acid and ethanol at reflux temperature resulted in an excellent yield of 85% of product 4a compared to other studied conditions (entry 2). The structure of 4a was thoroughly confirmed by nuclear magnetic resonance, infrared, and mass spectroscopy.

**TABLE 1:** Optimization of a model one-pot reaction to obtain 4a.^a^

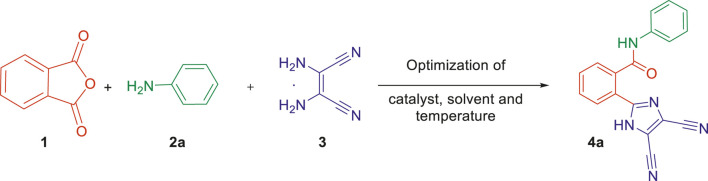					
Entry	Solvent	Temp (°C)	Catalyst	Time (h)	4a Yield (%)
1	Methanol	RT	HCl	12	78
2	Ethanol	RT	HCl	12	80
3	DMF	RT	HCl	8	70
4	DMSO	RT	HCl	8	70
5	Methanol	Reflux	HCl	3	80
6	Ethanol	Reflux	HCl	2	85
7	DMF	100	HCl	2	72
8	DMSO	100	HCl	2	72
9	Ethanol	Reflux	H_3_PO_4_	2	70
10	Ethanol	Reflux	CH_3_COOH	4	80
11	Ethanol	Reflux	HCl (0.2)	3	78
12	Ethanol	Reflux	HCl (1)	1.5	75

a Reaction was carried out with 1 mmol of substrates 1, 2a, and 3 in the presence of catalyst (0.5 equiv).

**SCHEME 1:**
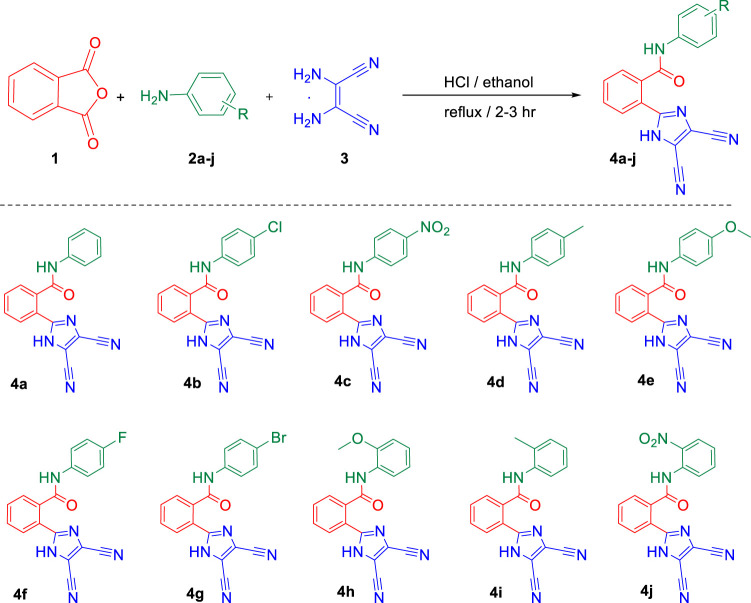
Synthesis of new imidazole-based *N*-phenylbenzamide derivatives 4a–j

Finally, after the optimization of the reaction conditions, we studied the applicability of the developed synthetic protocol with a series of various substituted anilines 2b–2j along with phthalic anhydride 1 and 2,3-diaminomaleonitrile 3 (Scheme 1). The developed one-pot multicomponent strategy provided the desired new imidazole-based *N*-phenylbenzamide derivatives (4a–j) with a good yield of 80–85%. It is interesting to note that the method was efficient toward both electron donation and withdrawing groups on the aniline and also the position of the groups on the aromatic ring.

### Anticancer Studies

The series of ten new imidazole-based *N*-phenylbenzamide derivatives, synthesized using a multicomponent strategy, was investigated for their cytotoxic potential against lung (A549), cervical cancer (HeLa), and breast (MCF-7) cancer cell lines. Most of the derivatives displayed good to moderate activity against the tested cell lines (Table 2). The derivative 4f, with a fluorine substitution, was the most active compound in the series, displaying single-digit anticancer activity. It exhibited IC_50_ values of 7.5, 9.3, and 8.9 µM against A549, HeLa, and MCF-7, respectively. Similarly, the derivative 4e with a para methoxy group also showed promising activity with IC_50_ values of 8.9, 11.1, and 9.2 µM against A549, HeLa, and MCF-7, respectively. It was interesting to note that the derivative 4h, with an orthomethoxy group, also displayed pronounced IC_50_ values in the range of 9.3–11.9 µM. A good to moderate activity was shown by the derivatives 4a, 4b, 4d, 4g, and 4i with IC_50_ values in the range of 11.6–45.3 µM. In contrast, the derivatives 4c and 4j showed little activity against the tested cancer cell lines.

**TABLE 2:** Cytotoxicity of new imidazole-based *N*-phenylbenzamide derivatives 4a–j.

Test compound	IC_50_ value in µM (mean ± SD)^a^		
	A549	HeLa	MCF-7
4a	23.1 ± 0.12	36.4 ± 0.28	23.5 ± 0.15
4b	20.9 ± 0.36	16.4 ± 0.32	19.1 ± 0.26
4c	>100	55.6 ± 0.44	73.2 ± 0.39
4d	14.7 ± 0.22	12.1 ± 0.16	14.6 ± 0.24
4e	8.9 ± 0.17	11.1 ± 0.15	9.2 ± 0.12
4f	7.5 ± 0.38	9.3 ± 0.11	8.9 ± 0.11
4g	13.4 ± 0.14	45.3 ± 0.39	22.3 ± 0.26
4h	10.7 ± 0.13	11.9 ± 0.12	9.3 ± 0.37
4i	16.9 ± 0.25	13.1 ± 0.27	11.6 ± 0.15
4j	>100	>100	69.5 ± 0.46
Dox	0.8 ± 0.05	0.7 ± 0.06	0.9 ± 0.08

a Experiments were carried out in triplicates. Doxorubicin (Dox) was used as a positive control.

A structure–activity insight was developed from the experimental results, revealing that the substituent’s position on the aromatic phenyl ring led to a pronounced enhancement in the activity. However, there was no noticeable differential effect exhibited by electron-donating and electron-withdrawing groups. In the case of electron-donating groups, the methyl and methoxy substituents at both ortho and para positions showed improvement in activity. The methoxy derivatives, 4e and 4h, were more active than the methyl derivatives. On the other hand, the derivatives containing electron-withdrawing groups showed varied results. The derivative 4f, with a para-substituted fluorine group, displayed the highest activity in the series. However, moving toward the lesser electronegative chloro and bromo groups, the activity decreased. Finally, it was noteworthy that the nitro substitution, irrespective of its position (as in derivatives 4c and 4j), led to a complete loss of activity.

### Computational Analysis

#### In Silico Binding Studies

**FIGURE 2:**
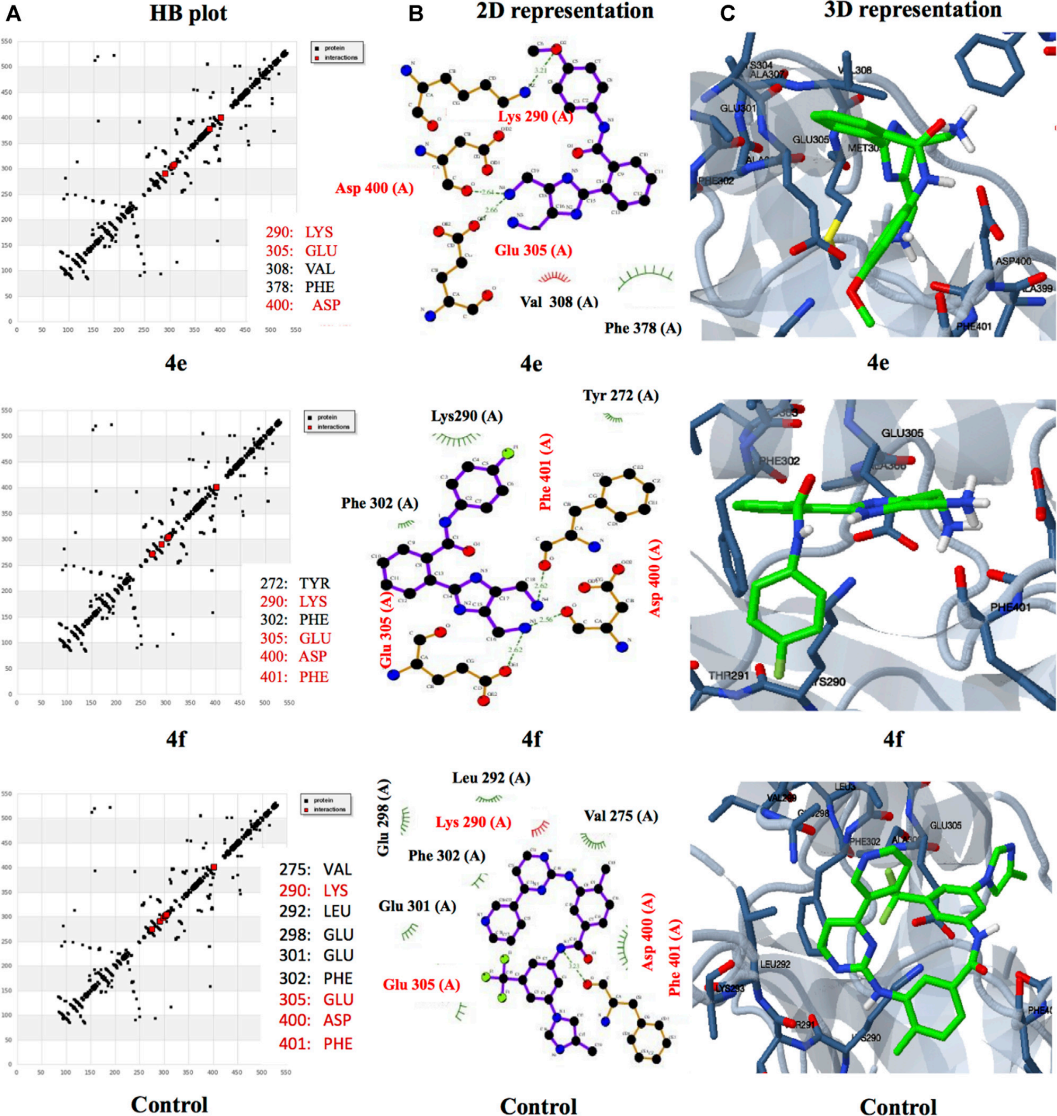
**(A)** HB plots showing hydrogen bonding interaction of different ligands 4e (290:LYS, 305:GLU, 308:VAL, 378:PHE, and 400:ASP), 4f (275:VAL, 290:LYS, 292:LEU, 298:GLU, 301:GLU, 302:PHE, 305:GLU, 400: ASP, 401:PHE) and control, nilotinib (275:VAL, 290:LYS, 292:LEU, 298:GLU, 301:GLU, 302:PHE, 305:GLU, 400:ASP, and 401:PHE) with common interactive residues shown in red. **(B)** Overview of the 2D interactions of ABL1 kinase with ligands 4e, 4f, and nilotinib. **(C)** 3D molecular interactions of different ligands 4e, 4f, and control interacting with TYL kinase (5MO4) in the binding pocket.

Abelson (ABL) tyrosine kinases have emerged as attractive targets in targeted anticancer therapies in recent years. A family member, ABL1, is well established to play a critical role in cancer progression, particularly in chronic myeloid leukemia (CML) (Lee et al., 2021). The structural similarity of our new imidazole-based *N*-phenylbenzamide derivatives to nilotinib, a clinically used ABL1 kinase inhibitor, encouraged us to undertake docking investigations to understand their interaction with ABL1 kinase at the molecular level. The active derivatives, 4e and 4f, were subjected to in silico docking to reveal the interaction with the kinase protein. The clinical drug nilotinib was used as a control (Figure 2). The results showed that the derivatives 4e and 4f exhibited excellent binding affinities toward the kinase protein, compared to nilotinib. The best binding affinities for derivatives 4e and 4f were −8.59 and −7.44 kcal/mol, respectively. On the other hand, the control nilotinib showed the best binding affinity of −5.75 kcal/mol.

A similar trend was observed with inhibition constant values as the derivatives showed better inhibition constant values than the control. The derivatives 4e and 4f showed inhibition constant values of 505.37 nM and 3.52 μM, respectively. In the case of control nilotinib, the inhibition constant value was 60.6 μM. The lower values of binding energy and inhibition constant suggest that the derivatives interact with the residues of ABL1 kinase spontaneously and could stabilize ligand–target protein complexes, leading to inhibition. The interaction of the ligands with the protein was through hydrogen bond formation and hydrophobic and electrostatic interactions (see Supplementary Information).

#### Molecular Dynamics Simulations

**FIGURE 3:**
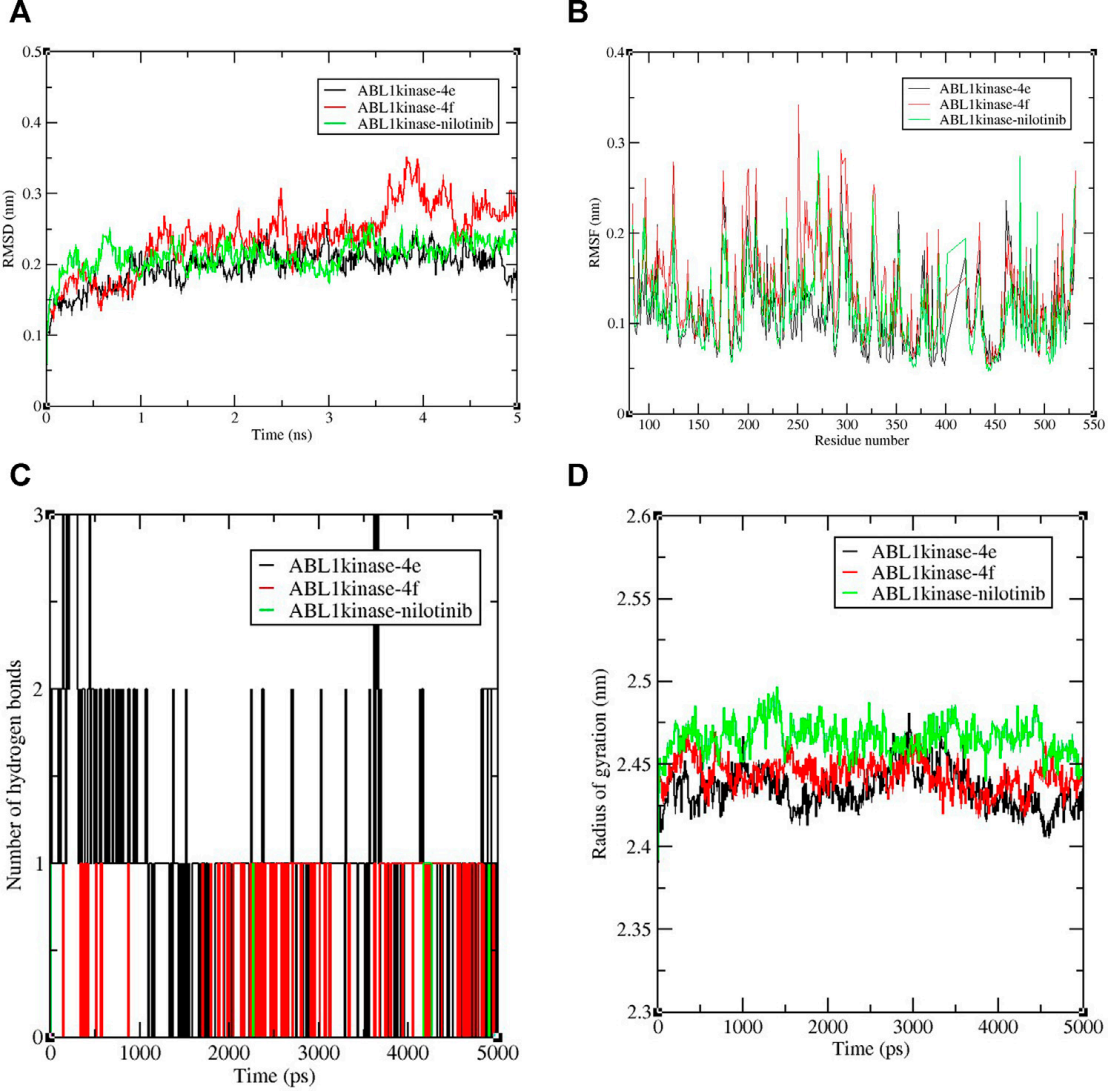
Graphical representation of **(A)** RMSD plot of ABL1 kinase-4e (black), ABL1 kinase-4f (red), and ABL1 kinase–nilotinib (green) complexes deviation during 5 ns period. **(B)**RMSF plot with fluctuation per residues of the complexes. **(C)** Hydrogen bond plot depicting formation hydrogen bond of the complexes during 5,000-ps period of simulation. **(D)** Radius of gyration (Rg) plot representing compactness of complexes during 5,000 ps simulation. nm = nanometer, ns= nanosecond, ps = picosecond.

Nowadays, molecular dynamic simulation (MDS) is an increasingly employed tool in drug discovery and development programs. MDS provides valuable insights into the thermodynamics and kinetic behavior of drug–target complexes (De Vivo et al., 2016). The excellent docking scores of derivatives 4e and 4f, compared to the control nilotinib, encouraged us to perform MD simulations to study and compare the binding stabilities of the protein-ligand complexes. Therefore, an MDS experimentation of 5-ns run analysis was performed, and the obtained data were analyzed for root-mean-square deviation (RMSD), root-mean-square fluctuation (RMSF), the radius of gyration, and formation of hydrogen bonds. The results revealed the deviation and fluctuation of ABL1 kinase-4e, ABL1 kinase-4f, and ABL1 kinase–nilotinib complexes during the complete simulation period. Overall, the observed RMSD values were between 0.1 and 0.3 nm for all complexes until 3.5 ns complexes and were stable with a value of 0.2 nm, except for the ABL1 kinase-4f complex which showed a slightly higher value around 0.3 nm until 5 ns. Interestingly, ABL1 kinase-4e showed a similar value of 0.2 nm compared to the control RMSD value of 0.2 nm (Figure 3A).

The RMSF calculation per residue value ranged between 0.1 and 0.2 nm (Figure 3B). Few fluctuations were observed at 100–125 and 250–300 amino acid residue regions. In the case of hydrogen bond analysis, the bond plot revealed the formation of 1–3 hydrogen bonds during the 5-ns period (Figure 3C). During the simulation, the radius of gyration analysis is critical in assessing the compactness and stability of the protein structure due to interaction with ligand molecules. The observed average values of the radius of gyration were between 2.4 and 2.5 nm. It was also shown that for most of the time, compactness was maintained with a gyration radius value around 2.45 nm (Figure 3D). Overall, the radius of gyration analysis suggested ABL1 kinase-4e and ABL1 kinase-4f complexes showed marginally less value than standard ABL1 kinase–nilotinib fluctuation during the 5,000-ps simulation.

#### ADME, Drug-Likeness Analysis

**FIGURE 4:**
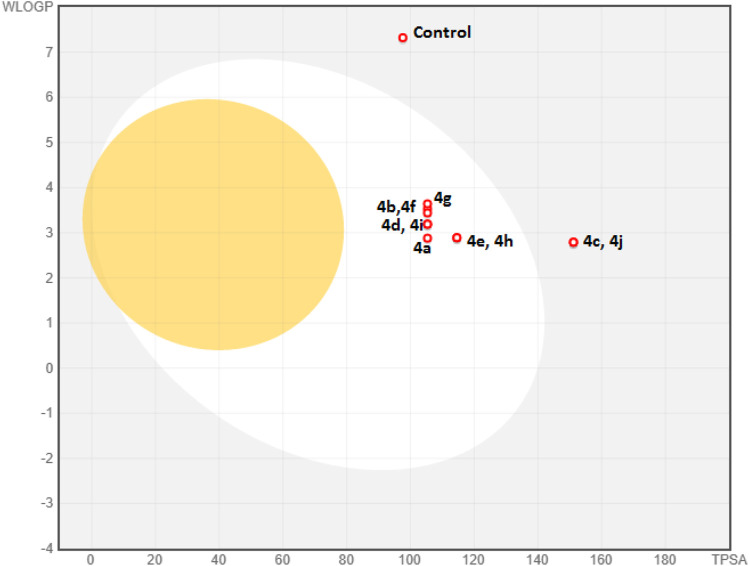
BOILED-Egg model of derivatives 4a–j and control (nilotinib). The yolk and the white in the model denote the BBB permeability and GI absorption, respectively. Blue dots represent the derivatives that are not effluated from CNS.

Computational pharmacodynamics and pharmacokinetics analyses help in understanding the behavior of drugs in the natural environment, thereby avoiding the costly experimental studies of all potential drugs (Alqahtani 2017). Interestingly, computational approaches interfacing chemistry and biology are being exploited to rationalize and repurpose traditional medicines (Adhikari et al., 2020). The good cytotoxic potential of the new derivatives 4a–j encouraged us to undertake their ADME and drug-likeness predictions. The metabolism of foreign compounds is carried out by the cytochrome P450 (CYP) family, and the inhibition of these enzymes leads to significant drug–drug interactions (Beck et al., 2021). All the new derivatives 4a–j showed a similar profile by inhibiting four isoforms (CYP1A2, CYP2C9, CYP2D6, and CYP3A2) and no inhibition of the CYP2C19 isoform (Table 3). In the case of the absorption aspect, the gastrointestinal (GI) absorption and the permeability through the blood–brain barrier (BBB) of the new derivatives (4a–4j) were predicted by a Brain Or IntestinaL EstimateD permeation (BOILED-Egg) model (Figure 4) (Daina and Zoete, 2016). The results revealed that all the derivatives, except with nitro substitution (4c and 4j) could be easily absorbed through the gastrointestinal tract. However, all the derivatives were not permeable through the blood–brain barrier and were predicted to be not effluated from CNS by p-glycoprotein. Interestingly, the control nilotinib was predicted to be neither absorbed by gastrointestinal tract nor permeable to the BBB. In addition to this, all the derivatives exhibited a bioactive score of 0.55 and a skin permeability coefficient (log Kp) in the range of −6.72 to −6.09 cm/s. Detailed information on ADME analysis is provided in the Supplementary Material.

**TABLE 3:** Pharmacokinetics and drug-likeness predictions of new imidazole-based *N*-phenylbenzamide derivatives 4a-j.

Compound	Inhibition of cytochromes			log Kp (cm/s)	TPSA value	Number of drug-likeness filter followed (out of 5)
	1A2	2C19	2C9, 2D6, 3A4			
4a	Y	N	Y	−6.32	105.36	5
4b	Y	N	Y	−6.09	105.36	5
4c	Y	N	Y	−6.72	151.18	2
4d	Y	N	Y	−6.15	105.36	5
4e	Y	N	Y	−6.53	114.59	5
4f	Y	N	Y	−6.36	105.36	5
4g	Y	N	Y	−6.31	105.36	5
4h	Y	N	Y	−6.53	114.59	5
4i	Y	N	Y	−6.15	105.36	5
4j	Y	N	Y	−6.33	151.18	2
Nilotinib	N	Y	Y	−6.05	97.62	2

The drug-likeness prediction tools help in addressing the high attrition rate in drug development programs (Agoni et al., 2020). The drug-likeness properties of potential agents are predicted by five primary filters, namely, Lipinski, Ghose, Veber, Egan, and Muegge. In the present study, the drug-likeness properties were predicted by computational tools, and excellent results were observed. Except with nitro substitution (4c and 4j), all the derivatives showed no violation of all the five filters. The derivatives 4c and 4j showed a single violation with Veber, Egan, and Muegge filters because of higher topological polar surface area (TPSA) values (see Supplementary Information). On the other hand, the control nilotinib showed the violation of primary Lipinski and Ghose filters along with the Egan filter.

## Conclusion

Cancer, being the disease of one’s cells, exhibits enormous challenges because of the high rate of cancer cases and the emergence of resistance toward clinically used drugs. This creates a perpetual demand for research endeavors to develop new anticancer agents. The *N*-phenylbenzamide derivatives and imidazole moieties are critical structural motifs in different anticancer drugs that are used in the clinic. Taking into consideration the usefulness of these structural motifs, we designed a series of new imidazole-based *N*-phenylbenzamide derivatives 4a–j using an atom-economical multicomponent approach. A cytotoxicity assay showed that all the derivatives, except two, showed promising anticancer potency. However, the derivatives 4f, with a para fluorine substituent, and 4e, with a para methoxy substituent, were the most active compounds with single-digit IC_50_ values. The molecular docking revealed that the derivatives 4e and 4f showed better binding affinity toward the ABL1 kinase receptor protein than the clinically used control drug, nilotinib. This prompted us to analyze the stability of ABL1 kinase and ligand (4e, 4f) complexes and compare it with that of the ABL1 kinase–nilotinib complex. Interestingly, the stability of the complexes formed between ABL1 kinase and ligands (4e, 4f) was almost similar to that of the ABL1 kinase–nilotinib complex. The ADME analysis revealed that most compounds have good gastrointestinal absorption properties, while the control showed no GI absorption. The drug-likeness predictions of the derivatives showed unique insights, and all of the derivatives (except two) followed all the five drug-likeness rules, that is, Lipinski, Ghose, Veber, Egan, and Muegge. The present study offers valuable insights and a good starting point to carry out advanced developmental studies of the most active derivatives 4e and 4f for further development of these useful potential anticancer agents.

## Data Availability

The original contributions presented in the study are included in the article/Supplementary Material; further inquiries can be directed to the corresponding authors.
