# Supplementary material for: New Imidazole-Based N-Phenylbenzamide Derivatives as Potential Anticancer Agents: Key Computational Insights
Source: Front Chem. 2022 Jan 19;9:808556. doi: 10.3389/fchem.2021.808556 (PMC8830504; doi:10.3389/fchem.2021.808556)
Supplement: Supplementary file 1 [file DataSheet1.PDF]

Supporting information

New imidazole-based *N*-phenylbenzamide derivatives as potential anticancer agents: Key computational insights

M. Shaheer Malik[1]*, Reem I. Alsantali[2], Qazi Mohammad Sajid Jamal[3], Zaki S. Seddigi[4], Moataz Morad[1], Meshari A. Alsharif[1], Essam M. Hussein[1,5], Rabab S. Jassas[6], Munirah M. Al-Rooqi[1], Zainularifeen Abduljaleel[7], Ahmed O. Babalgith[8], Hatem M. Altass[1], Ziad Moussa[9], Saleh A. Ahmed[1,5]*

Contents:

- Molecular docking of derivative **4e**, **4f** and control with ABL1 kinase: Values of different parameters
- Molecular interaction of ligands **4e**, **4f** and control with ABL1 kinase protein
- Prediction of physiochemical properties of the derivatives **4a-j** and control
- Prediction of drug likeness properties of the derivatives **4a-j** and control

Table: 1

Molecular docking of derivative **4e**, **4f** and control with ABL1 kinase: Values of different parameters

Complex with Control	Rank	Est. Free Energy of Binding	Est. Inhibition Constant, Ki	vdW + Hbond + desolv Energy	Electrostatic Energy	Total Intermo lec. Energy	Frequency	Interact. Surface
ABL1kinase + Nilotinib ©	1	-5.75 kcal/mol	60.62 uM	-6.67 kcal/mol	+0.07 kcal/mol	-6.60 kcal/mol	50%	865.323
	2	-1.17 kcal/mol	138.19 mM	-2.15 kcal/mol	-0.08 kcal/mol	-2.23 kcal/mol	50%	707.901

Complex with Ligand	Rank	Est. Free Energy of Binding	Est. Inhibition Constant, Ki	vdW + Hbond + desolv Energy	Electrostatic Energy	Total Intermo lec. Energy	Frequency	Interact. Surface
ABL1kinase + 4f	1	-7.44 kcal/mol	3.52 uM	-5.66 kcal/mol	-3.07 kcal/mol	-8.73 kcal/mol	50%	622.145
	2	-6.76 kcal/mol	11.16 uM	-6.32 kcal/mol	-0.83 kcal/mol	-7.16 kcal/mol	50%	710.082

Complex with Ligand	Rank	Est. Free Energy of Binding	Est. Inhibition Constant, Ki	vdW + Hbond + desolv Energy	Electrostatic Energy	Total Intermo lec. Energy	Frequency	Interact. Surface
ABL1kinase + 4e	1	-8.59 kcal/mol	505.37 nM	-5.58 kcal/mol	-3.57 kcal/mol	-9.15 kcal/mol	50%	717.545
	2	-6.35 kcal/mol	22.06 uM	-4.01 kcal/mol	-2.67 kcal/mol	-6.68 kcal/mol	50%	698.798

Table: 2. Molecular interaction of ligands **4e**, **4f** and control with ABL1 kinase protein

Decomposed Interaction Energies in kcal/mol (Control: nilotinib)					
**Hydrogen bonds**	**Cation-pi**	**Halogen-bond**	**Polar**	**Hydrophobic**	**Other**
PHE401 (-0.9313)		GLU305 (-1.3561)	ASP400 (-0.981)	LEU292 (-0.3598)	PHE302 (-1.6059)
		GLU301 (-0.6808)	LYS290 (0.491)	VAL275 (-0.3253)	GLU298 (-0.5834)

Decomposed Interaction Energies in kcal/mol (Ligand: 4F)					
**Hydrogen bonds**	**Cation-pi**	**Halogen-bond**	**Polar**	**Hydrophobic**	
PHE401 (-40.396)		TYR272 (-0.162)	LYS290 (-33.581)	PHE302 (-4.4438)	
ASP400 (41.1912)					
GLU305 (100.287)					

Decomposed Interaction Energies in kcal/mol (Ligand: 4E)					
**Hydrogen bonds**	**Cation-pi**			**Hydrophobic**	
LYS290 (-0.7042)	PHE378 (-18.077)			VAL308 (2.8218)	
GLU305 (-0.3747)					
ASP400 (3.2503)					

Table 3: Prediction of physiochemical properties of the derivatives **4a-j** and control, nilotinib.

**Compounds**	**No. of Rotatable bonds**	**No. of H-bond acceptors**	**No. of H-bond donors**	**Molecular refraction**	**iLOGP**	**XLOGP3**	**WLOGP**
**4a**	4	4	2	87.67	1.37	2.66	2.88
**4b**	4	4	2	92.68	1.54	3.29	3.53
**4c**	5	6	2	96.49	1.38	2.49	2.79
**4d**	4	4	2	92.63	1.85	3.03	3.19
**4e**	5	5	2	94.16	1.78	2.63	2.89
**4f**	4	5	2	87.63	1.52	2.76	3.44
**4g**	4	4	2	95.37	1.42	3.35	3.64
**4h**	5	5	2	94.16	2.89	2.63	2.89
**4i**	4	4	2	92.63	1.54	3.03	3.19
**4j**	5	6	2	96.49	1.42	3.04	2.79
**Control**	8	8	2	141.08	3.36	4.9	7.32

Table 3: Prediction of drug likeness properties of the derivatives **4a-j** and control, nilotinib

Compound	No of Lipinski violations	No. of Ghose violations	No. of Veber violations	No. of Egan violations	No. of Muegge violations	Bioavailability Score	No. of PAINS alerts	No. of Brenk alerts	No of Leadlikeness violations	Synthetic Accessibility
**4a**	0	0	0	0	0	0.55	0	0	0	2.42
**4b**	0	0	0	0	0	0.55	0	0	0	2.44
**4c**	0	0	1	1	1	0.55	0	2	1	2.61
**4d**	0	0	0	0	0	0.55	0	0	0	2.53
**4e**	0	0	0	0	0	0.55	0	0	0	2.49
**4f**	0	0	0	0	0	0.55	0	0	0	2.42
**4g**	0	0	0	0	0	0.55	0	0	1	2.5
**4h**	0	0	0	0	0	0.55	0	0	0	2.56
**4i**	0	0	0	0	0	0.55	0	0	0	2.55
**4j**	0	0	1	1	1	0.55	0	2	1	2.82
**Control**	1	3	0	1	0	0.55	0	0	3	3.81
